# When the CAT wants to play: The role of interaction between CRCK3 and CAT2 in Arabidopsis salt stress tolerance

**DOI:** 10.1093/plphys/kiaf050

**Published:** 2025-02-05

**Authors:** Sara Selma

**Affiliations:** Assistant Features Editor, Plant Physiology, American Society of Plant Biologists, Rockville, MD 20855-2768, USA; VIB Center for Plant Systems Biology, 9052 Ghent, Belgium

Climate change affects multiple global processes, particularly impacting agriculture and food production. Soil salinization, which severely compromises crop productivity in cultivable areas, is one of the most pressing environmental challenges. The accumulation of salts in the soil diminishes water availability for plants and alters the cellular balance, leading to metabolic and growth issues. The salt stress also increases the reactive oxygen species (ROS) produced in the cell, which causes severe oxidative damage at a molecular level, threatening plant health and survival (Zhou et al. 2024).

To deal with oxidative damage, plants have developed finely tuned mechanisms to manage ROS production, such as producing several ROS detoxifying enzymes, including catalases (CATs). In *Arabidopsis thaliana* (Arabidopsis) plants, 3 catalases, CAT1, CAT2, and CAT3, have been identified, with CAT2 being the most active enzyme among the group (Du et al. 2008). The triple mutation of the CAT genes in Arabidopsis severely compromises the plant's resistance to salt stress compared with wild-type plants (Su et al. 2018). This observation highlights the importance of CAT proteins in dealing with the oxidative stress caused by the increasing salt in the environment. For that reason, identifying the players involved in regulating CAT activity is crucial for understanding oxidative defenses in plants and improving crop resilience (Yang and Guo 2018; Zhang et al. 2021; Baker et al. 2023).

In this issue of *Plant Physiology*, Zhuang et al. uncovered the role of the RLCK CALMODULIN-BINDING RECEPTOR-LIKE CYTOPLASMIC KINASE 3 (CRCK3), a calcium-dependent protein kinase, in plant salt tolerance through its interaction with CAT2 in Arabidopsis.

Previously, the authors identified the 3 Arabidopsis CRCK proteins—CRCK1, CRCK2, and CRCK3—as potential regulators of salt tolerance (Zhang et al. 2023). The salt-responsive expression of the *CRCK* genes was demonstrated by treating wild-type plants with 150 mm NaCl to trigger salt stress, followed by RT-qPCR. CRCK-GUS reporter lines showed different patterns of accumulation of the different *CRCK* genes, suggesting that the CRCK family members can play different roles in plant salt stress response.

Arabidopsis mutant lines of *CRCK1*, *CRCK2*, and *CRCK3* were analyzed in salt stress conditions. Only the *crck3* mutants showed severe salt sensitivity, with delayed germination, reduced accumulation of chlorophyll, and inhibited root and shoot growth when exposed to salt. Furthermore, *crck3* plants exhibited greater photoinhibition and lower expression of salt-responsive genes such as *RD22* and *KIN2* (Wang et al. 2022), confirming the crucial role of CRCK3 in salt tolerance in Arabidopsis.

To investigate whether the activity of the CRCK3 protein under salt stress is dependent on its kinase activity, the authors generated 2 *crck3* complementation lines: one with a functional CRCK3 (COM) and one with a mutated version (mCOM) lacking kinase activity. The COM line rescued the salt sensitivity of *crck3* plants, while the mCOM did not rescue the phenotype. Additionally, stress-responsive genes like *RD22* and *KIN2* were upregulated in the COM plants under salt stress but not in the mCOM plants.

Co-immunoprecipitation assays followed by mass spectrometry identified the catalase family proteins (CAT1, CAT2, and CAT3) as potential CRCK3-interacting partners. BiFC assays confirmed that CRCK3 interacts with CATs, particularly CAT2, through the kinase domain of CRCK3. This interaction with CAT2 points to a direct role of CRCK3 in maintaining redox homeostasis during salt stress. The authors evaluated CAT activity in *crck3* and CRCK3-mCOM mutant lines and observed a reduced activity of CAT2 in both mutants and a high ROS accumulation under salt stress compared with wild-type or CRCK3-COM lines, which was further supported by higher oxidized protein levels and increased ROS-induced gene expression in the mutants.

To investigate the functional relationship between catalases and CRCK3, the authors generated a collection of catalase mutant lines and overexpression lines. The *cat2* mutant line was more salt-sensitive than the wild type, while in the *cat3* mutant line, this effect was not observed; previous studies had shown that CAT2 is the most highly expressed CAT. The *cat2cat3crck3* triple mutant showed a similar salt-sensitive phenotype compared with the *cat2cat3* lines, indicating that CAT2 is downstream of CRCK3 in the salt response, whereas overexpression of CAT2 in *crck3* plants partially rescued the salt-sensitive phenotype observed in *crck3* lines. On the other hand, overexpression of CRCK3 in the *cat2cat3* mutant did not rescue the salt-sensitive phenotype, further suggesting that CRCK3 mediates salt tolerance in Arabidopsis through its effect on CAT2.

Finally, the authors looked at a putative phosphorylation target in CAT2, Thr209. They looked at transgenic plants expressing different versions of CAT2 in *cat2* background plants: a wild-type CAT2 (35Spro::CAT2-GFP), a nonphosphorylatable mutant (35Spro::CAT2T209A-GFP), and a phosphomimetic mutant (35Spro::CAT2T209D-GFP). The nonphosphorylatable mutant did not rescue the salt sensitivity, unlike the wild-type CAT2. On the other hand, the phosphomimetic version of CAT2 fully rescued the salt sensitivity indicating that phosphorylation of CAT2 at Thr209 is crucial for salt tolerance.

In summary, the authors showed that salt exposure mediates the activation of CRCK3, resulting in its interaction and phosphorylation of the CAT2 protein, leading to enhanced ROS detoxification activity under salt stress (Figure). This work demonstrates that CRCK3 plays a central role in regulating plant salt tolerance. This pathway is crucial for maintaining redox balance and mitigating oxidative damage generated during soil salinization. These findings can be used to help to generate more resilient crops in an increasingly hostile environment.

**Figure.**
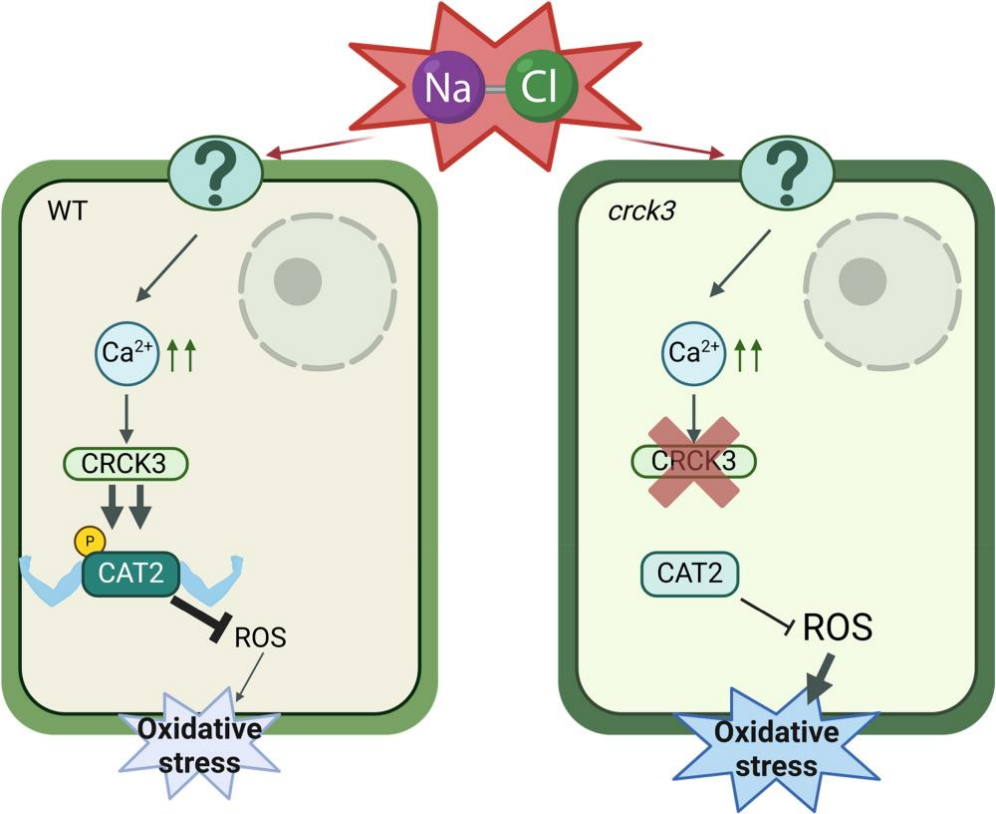
Model of action of CRCK3-CAT2 interaction under salt stress in Arabidopsis. In wild-type plants, salt stress triggered Ca^2+^ in the cytoplasm, which activates CRCK3. The activated CRCK3 interacts and phosphorylates CAT2 at Thr209, increasing CAT2 activity to scavenge ROS and reducing oxidative stress in the plant. On the other side, in the mutant *crck3* plants, the CAT2 cannot be fully activated in response to salt stress, thus increasing the level of ROS and generating severe oxidative stress. Image adapted from Zhuang et al. (2025) and created by Biorender.com.
